# Luteolin promotes apoptotic cell death via upregulation of Nrf2 expression by DNA demethylase and the interaction of Nrf2 with p53 in human colon cancer cells

**DOI:** 10.1038/s12276-019-0238-y

**Published:** 2019-04-15

**Authors:** Kyoung Ah Kang, Mei Jing Piao, Yu Jae Hyun, Ao Xuan Zhen, Suk Ju Cho, Mee Jung Ahn, Joo Mi Yi, Jin Won Hyun

**Affiliations:** 1 Jeju National University School of Medicine and Jeju Research Center for Natural Medicine, Jeju, 63243 Republic of Korea; 2 Laboratory of Veterinary Anatomy, College of Veterinary Medicine, Jeju National University, Jeju, 63243 Republic of Korea; 3 Department of Microbiology and Immunology, Inje University College of Medicine, Busan, 47392 Republic of Korea

**Keywords:** Colon cancer, Apoptosis

## Abstract

Luteolin, a dietary flavone, modulates various signaling pathways involved in carcinogenesis. In this study, we investigated the molecular mechanism that underlies the apoptotic effects of luteolin mediated by DNA demethylation of the nuclear factor erythroid 2-related factor 2 (Nrf2) promoter and the interaction of Nrf2 and p53, a tumor suppressor, in human colon cancer cells. Luteolin increased the expression of apoptosis-related proteins and antioxidant enzymes. In DNA methylation, luteolin inhibited the expression of DNA methyltransferases, a transcription repressor, and increased the expression and activity of ten-eleven translocation (TET) DNA demethylases, a transcription activator. Methyl-specific polymerase chain reaction and bisulfite genomic sequencing indicated that luteolin decreased the methylation of the Nrf2 promoter region, which corresponded to the increased mRNA expression of Nrf2. In addition, luteolin increased TET1 binding to the Nrf2 promoter, as determined using a chromatin immunoprecipitation (ChIP) assay. TET1 knockdown decreased the percentages of luteolin-treated cells in sub-G_1_ phase and cells with fragmented nuclei. Furthermore, complex formation between p53 and Nrf2 was involved in the apoptotic effects of luteolin. These results provide insight into the mechanism that underlies the anticancer effects of luteolin on colon cancer, which involve the upregulation of Nrf2 and its interaction with the tumor suppressor.

## Cancer: Cell-killing plant compound exerts antioxidant effects

A molecule found in fruits, vegetables and herbs helps kill colon cancer cells by activating a master regulator of detoxifying enzymes. Jin Won Hyun from Jeju National University School of Medicine in South Korea and colleagues treated human colon cancer cells with luteolin, a molecule that occurs naturally in many food plants. They showed that luteolin increased the levels of proteins involved in cell death and antioxidant responses by causing DNA-modifying enzymes to strip suppressive chemical markers off the gene encoding Nrf2, a protein that regulates antioxidant effects. Nrf2 levels subsequently increased and the protein interacted with the tumor suppressor p53 to facilitate destruction of the colon cancer cells. The findings offer a mechanistic basis for using luteolin to help prevent and treat cancer.

## Introduction

Colon cancer is a major cause of morbidity and mortality worldwide^1^. The accumulation of genetic and epigenetic alterations in the normal colon leads to the transformation of the normal colonic epithelium into colon adenocarcinomas^2^. Accumulating evidence indicates that DNA methylation of gene promoters increases in the colonic mucosa of colon cancer patients^3,4^. Aberrant DNA methylation is an important driving factor for colon cancer progression and metastasis. For example, *CXCL12*, which encodes a chemokine ligand, is aberrantly methylated in colon cancer patients; this aberrant methylation promotes the metastatic behavior of colon cancer cell lines^5^. In addition, multiple inactive genes, including *TIMP3*, *ID4*, and *IRF8*, with methylated promoter regions provide a clonal growth advantage, which results in more severe malignant phenotypes^5,6^.

Colon cancer originates from epithelial cells that line the bowel. These cells rapidly divide and have a high metabolic rate, which may increase DNA oxidation^7^. The levels of various oxidative stress markers, including reactive oxygen species (ROS), nitric oxide, 8-oxoguanine in DNA, and lipid peroxides, increase in human colon cancers such as adenomas and carcinomas^7^.

Nuclear factor erythroid 2-related factor 2 (Nrf2) helps protect cells against oxidative stress by regulating various phase II detoxifying enzymes, including antioxidant enzymes. Combination therapy with rapamycin and *S*-allylmercapto-cysteine reportedly has an enhanced cancer-suppressing ability by triggering apoptosis via the inhibition of autophagic activity and activation of Nrf2^8^. Furthermore, Nrf2-knockout mice are more susceptible to oxidative stress-induced diseases and chemical-induced DNA damage than wild-type mice, which increases their risk of certain types of cancer, such as those of the stomach, colon, and skin^9–11^. In a previous study, the incidence of cancer was higher in azoxymethane-treated Nrf2-knockout mice (80%) than in azoxymethane-treated wild-type mice (29%). Moreover, the expression of inflammatory markers, such as cyclooxygenase-2, 5-lipoxygenase, prostaglandin E_2_, and leukotriene B_4_, increased in the cancer tissues and inflamed colonic mucosa of Nrf2-knockout mice^12^.

Luteolin, a dietary flavone derived from vegetables, fruits, and herbs, has beneficial effects, including anti-inflammatory, anti-allergic, anticancer, and antioxidant, to prevent degenerative diseases^13–15^. It inhibits critical events associated with carcinogenesis, including cell invasion, metastasis, transformation, and angiogenesis, by inhibiting transcription factors, kinase modification, and cell cycle arrest and inducing apoptosis^16–18^.

Epigenetic modifications may underlie the anticancer activity of luteolin. Recently, Zao et al. demonstrated that luteolin epigenetically activates the Nrf2 pathway by downregulating DNA methyltransferase (DNMT) and histone deacetylase (HDAC) expression^19^. This study was aimed at investigating the apoptotic effects of luteolin on human colon cancer cells and elucidating the underlying mechanism involving DNA demethylase, ten-eleven translocation (TET) of the Nrf2 promoter, and the relationship between Nrf2 and p53, a tumor suppressor.

## Materials and methods

### Reagents

Luteolin, 3-(4,5-dimethylthiazol-2-yl)-2,5-diphenyltetrazolium bromide (MTT), Hoechst 33342, 2′,7′-dichlorofluorescein diacetate (DCF-DA), a mouse/rabbit red starter duolink kit, and propidium iodide (PI) were purchased from Sigma-Aldrich Corporation (St. Louis, MO, USA). Luteolin was prepared in 100% dimethyl sulfoxide to obtain a 100 mg/ml stock solution, which was aliquoted and stored at −20 °C for 3–6 months for use in the various experiments. Primary antibodies against p53, p21, Bcl-2, Bax, caspase-9, glutamate cysteine ligase catalytic (GCLc), glutathione synthetase (GSS), catalase, heme oxygenase-1 (HO-1), Nrf2, TET1, TET2, TET3, DNMT3B, and actin were purchased from Santa Cruz Biotechnology (Santa Cruz, CA, USA). DNMT1, phospho-Nrf2, and TATA box-binding protein (TBP) were purchased from Abcam (Cambridge, MA, USA), and caspase-3 and DNMT3A were purchased from Cell Signaling Technology (Beverly, MA, USA).

### Cell culture

Human HT-29 colon cancer and SNU-407 cells were obtained from the Korean Cell Line Bank (Seoul, Republic of Korea). Normal human FHC colon cells were obtained from the American Type Culture Collection (Rockville, MD, USA). HT-29 and SNU-407 cells were cultured in RPMI-1640 medium (Invitrogen, Grand Island, NY, USA) that contained 10% heat-inactivated fetal bovine serum (Sigma-Aldrich Co.). FHC cells were cultured in a 1:1 mixture of Ham’s F12 and DMEM that contained HEPES (25 mM), cholera toxin (10 ng/ml), insulin (5 μg/ml), transferrin (5 μg/ml), hydrocortisone (100 ng/ml), and 10% fetal bovine serum.

### Cell viability assay

The cells were seeded into 96-well plates at a density of 1 × 10^5^ cells/ml and treated with various concentrations of luteolin and 3.5 μM 5-aza-2′-deoxycytidine (5-aza-dC). After 48 h, 50 μl of MTT stock solution (2 mg/ml) was then added to each well to obtain a total reaction volume of 200 μl. After incubation for 4 h, the plate was centrifuged at 800 × *g* for 5 min, and the supernatants were aspirated. Formazan crystals in each well were dissolved in 150 μl of dimethylsulfoxide, and the absorbance was read at 540 nm using a scanning multi-well spectrophotometer^20^.

### Hoechst 33342 assay

Cells were seeded at a density of 1 × 10^5^ cells/ml, incubated at 37 °C for 24 h, and treated with 30 μM luteolin or 3.5 μM 5-aza-dC at 37 °C for an additional 48 h. The DNA-specific fluorescent dye Hoechst 33342 (1.5 μl, 10 mg/ml) was added to each well, and the cells were incubated for 10 min at 37 °C. Stained cells were visualized using a fluorescence microscope equipped with a CoolSNAP-Pro color digital camera (Media Cybernetics, Silver Spring, MD, USA).

### Western blot analysis

Cells were seeded at a density of 1 × 10^5^ cells/ml, incubated at 37 °C for 24 h, and treated with 10, 30, and 60 μM luteolin for 48 h or 30 μM for various times. The cells were harvested, washed twice with phosphate-buffered saline, lysed on ice for 30 min in 100 μl of lysis buffer (120 mM NaCl, 40 mM Tris [pH 8], and 0.1% NP 40), and centrifuged at 10,000 × *g* for 15 min. The supernatants were collected, and the protein concentrations were determined using a Bio-Rad protein assay reagent kit (Bio-Rad, Richmond, CA, USA). Protein lysates (40 μg) were electrophoresed and transferred onto nitrocellulose membranes, which were incubated with antibodies against p53, p21, Bcl-2, Bax, caspase-9, caspase-3, GCLc, GSS, catalase, HO-1, TET1, TET2, TET3, DNMT1, DNMT3A, DNMT3B, Nrf2, phospho-Nrf2, TBP, and actin. The membranes were subsequently incubated with secondary IgG conjugated with horseradish peroxidase (Pierce, Rockford, IL, USA). TBP was used as a loading control for nuclear proteins, while actin was the loading control for total and cytosolic proteins. Protein bands were detected using an enhanced chemiluminescence western blotting detection kit (Amersham, Little Chalfont, UK) and visualized using a luminescence image analyzer.

### Detection of ROS

ROS in cells were detected using flow cytometry after staining with DCF-DA (Sigma-Aldrich Co.)^21^. The cells were seeded in six-well plates at a density of 3 × 10^5^ cells/well, cultured for 24 h at 37 °C, pre-treated with various concentrations of luteolin for 1 h, and then treated with hydrogen peroxide (H_2_O_2_) for 24 h. Finally, the cells were treated with 25 µM DCF-DA, incubated for 10 min, and trypsinized, and the DCF fluorescence was analyzed using a flow cytometer at excitation and emission wavelengths of 485 and 535 nm, respectively (Becton Dickinson, Mountain View, CA, USA) and CellQuest™ software (Becton Dickinson).

### Detection of sub-G_1_ hypodiploid cells

Cells were seeded at a density of 1 × 10^5^ cells/ml, incubated at 37 °C for 24 h, and treated with 30 μM luteolin or 3.5 μM 5-aza-dC at 37 °C for an additional 48 h. Harvested cells were fixed in 70% ethanol for 30 min at 4 °C and incubated for 30 min in the dark at 37 °C in 1 ml of PBS that contained 100 μg of PI and 100 μg of RNase A. Flow cytometric analysis was performed using a FACSCalibur flow cytometer (Becton Dickinson). The percentage of sub-G_1_ hypodiploid cells was determined from histograms generated using the computer programs Cell Quest and Mod-Fit (Becton Dickinson).

### Measurement of 5-hydroxymethylcytosine (5-hmC)

The cells were seeded at 1.5 × 10^3^ cells/well in a four-well chamber slide (Thermo Fisher, Scoresby, Victoria, Australia) and treated with 30 μM luteolin for 12 h. After washing with PBS solution (PBS, 1 mM CaCl_2_, and 1 mM MgCl_2_) three times, the cells were fixed with cold 3% paraformaldehyde (PFA) for 15 min at 20 °C. The fixed cells were subsequently washed with 50 mM NH_4_Cl to quench the PFA followed by PBS solution and permeabilized with 0.1% saponin in PBS solution for 15 min at 20 °C. After permeabilization, the cells were incubated with a 5-hmC antibody diluted in PBS that contained 3% BSA for 2 h. The primary antibody was detected by staining with an Alexa Fluor 594-conjugated secondary antibody (1:500, Santa Cruz Biotechnology) for 1 h. Stained cells were mounted onto microscope slides in mounting medium that contained DAPI (Vector Laboratories, Burlingame, CA, USA) and imaged using a confocal microscope.

### Bisulfite sequencing analysis and quantitative methylation specific-PCR

For the methylation analysis, MethPrimer (http://www.urogene.org/methprimer/) was used to design primers that target the human Nrf2 promoter, which contains numerous CpG sites around the transcription start site. Amplicons for the bisulfite sequencing region (from −255 to −70 bp) and the methylation-specific PCR (MSP) region (from −313 to −166 bp) were designed from transcriptional start sites (TSS). To quantify the methylation level of the Nrf2 promoter region, bisulfite-treated genomic DNA samples were subjected to MS-qPCR, and the level of amplification was normalized against that of the Alu element^22^. qPCR was performed using the CFX96^TM^ system (Bio-Rad). To confirm the methylation level in the Nrf2 promoter sequence, we performed the bisulfite sequencing analysis. PCR amplicons were electrophoresed on a 2% agarose gel and purified using a gel extraction kit (Qiagen GmbH, Hilden, Germany). The amplicons were cloned into the TOPO TA vector system (Invitrogen). DNA was isolated and purified from individual clones using a NucleoSpin plasmid isolation kit (Macherey-Nagel, Düren, Germany). The M13F primer was used to sequence randomly selected positive clones (10–15 per sample), and the methylation status of each CpG dinucleotide was analyzed. All primers are listed in Table 1.

**Table 1:** Primer information for methylation analyses of Nrf2

		Primer sequences (5′–3′)	
		Forward	Reverse
Nrf2	Unmethylation	GTGGGTAATATTGATTATTTTTTGA	AATATAAACAACTCCAACAACTCATA
	Methylation	GGGTGGGTAATATTGATTATTTTTC	ATATAAACAACTCCGACAACTCGTA
	Bisulfite sequencing	ATTTGAGTTTAGGAGAATGGAGATA	AAAACTAAAAATTTAAACCCAAACC

### Real-time PCR

Cells were seeded at a density of 1 × 10^5^ cells/ml, incubated at 37 °C for 24 h, and treated with 30 μM luteolin at 37 °C for 0–48 h. Total RNA was isolated using Trizol (GibcoBRL, Grand Island, NY, USA). cDNA was prepared from 0.3 to 4 μg of RNA using Moloney murine leukemia virus (MMLV) reverse transcriptase and the oligo dT primer (Invitrogen). cDNA was mixed with 1 × Power SYBR Green PCR Master Mix (Applied Biosystems, Foster City, CA, USA) and forward and reverse primers (300 nM). The following primers were used: Nrf2, forward, 5′-GAGAGCCCAGTCTTCATTGC-3′, and reverse, 5′-TTGGCTTCTGGACTTGGAAC-3′; and actin, forward, 5′-CACCAACTGGGACGACAT-3′, and reverse, 5′-ACAGCCTGGATAGCAACG-3′. The PCR conditions were as follows: 95 °C for 10 min, followed by 40 cycles of 95 °C for 15 s and 58 °C for 1 min. PCR was performed in a 96-well plate using a Bio-Rad iQ5 real-time PCR detector system (Bio-Rad).

### ChIP assay

Cells were seeded at a density of 1 × 10^5^ cells/ml, incubated at 37 °C for 24 h, and treated with 30 μM luteolin at 37 °C for 48 h. The chromatin immunoprecipitation **(**ChIP) assay was performed using a simple ChIP enzymatic ChIP kit (Cell Signaling Technology) according to the manufacturer’s protocol with slight modifications. Briefly, the cells were cross-linked by the addition of 1% formaldehyde. Chromatin was prepared and digested with nuclease for 12 min at 37 °C. ChIP was performed with antibodies against TET1, DNMT1, and IgG. The antibodies were added to chromatin digests and incubated with constant rotation overnight at 4 °C. ChIP-grade protein G magnetic beads were subsequently added to capture the immune complexes. The beads were washed, and the immunoprecipitates were eluted with ChIP elution buffer. The cross-links were reversed by incubation at 65 °C for 30 min. Proteinase K was added, and the samples were incubated at 65 °C for 2 h. The immunoprecipitated DNA fragments were purified using spin columns. DNA recovered from the immunoprecipitated complex was subjected to 35 cycles of PCR. The primers that targeted the DNMT1- and TET1-binding sites of Nrf2 were as follows: forward, 5′-TGAGATATTTTGCACATCCGATA-3′, and reverse, 5′-ACTCTCAGGGTTCCTTTACACG-3′. The PCR products were separated on 2% agarose gels, and DNA bands were visualized.

### RNA interference

Cells were seeded at a density of 1 × 10^5^ cells/well in six-well plates and allowed to reach approximately 50% confluence on the day of transfection. Cells were transfected with 10–50 nM of a mismatched siRNA control (siControl) or siRNAs against TET1, p53, and p21 (siTET1 RNA No. 1044506, sip53 RNA No. 7157 and sip21 RNA No. 1026, Bioneer, Daejeon, Republic of Korea) using Lipofectamine RNAiMax (Invitrogen) according to the manufacturer’s instructions. At 24 h after transfection, the cells were examined by western blotting.

### IP assay

Cells were seeded at a density of 1 × 10^5^ cells/ml, incubated at 37 °C for 24 h, and treated with 30 μM luteolin for 12 or 24 h. Nuclear fraction lysates were incubated overnight at 4 °C with Nrf2, p53, or IgG antibody. Immune complexes were collected with protein A/G PLUS-beads (Santa Cruz Biotechnology) overnight at 4 °C and washed with IP buffer. Equal amounts of the immunoprecipitates were electrophoresed on SDS-polyacrylamide gel. Western blotting was performed using antibodies specific for Nrf2 and p53.

### Proximity ligation assay

A mouse/rabbit red starter duolink kit (Sigma-Aldrich Co.) was used. The cells were seeded at a density of 1.5 × 10^3^ cells/well in a 4-well chamber slide and treated with 30 μM luteolin for 12 or 24 h. After washing with PBS solution (1 mM CaCl_2_ and 1 mM MgCl_2_) three times, the cells were fixed with cold 3% paraformaldehyde (PFA) for 15 min at 20 °C. The fixed cells were subsequently washed with 50 mM NH_4_Cl to quench the PFA followed by a PBS solution wash and permeabilized with 0.1% saponin in PBS solution for 15 min at 20 °C. After permeabilization, the cells were incubated in the blocking buffer (provided with the kit) overnight at 37 °C in a humidified chamber and then incubated with primary antibodies: rabbit anti-Nrf2 (1:500) and mouse anti-p53 (1:500) diluted in fluorescence dilution buffer (5% fetal calf serum, 5% normal donkey serum, and 2% bovine serum albumin in PBS solution, pH 7.6) for 2 h at 20 °C. For the rest of the protocol, the manufacturer’s instructions were followed. Briefly, the cells were washed in buffer A (supplied with the kit) three times for 15 min and incubated with proximity ligation assay (PLA) probes for 1 h at 37 °C in a humid chamber. This step was followed by a 10 min and 5 min wash in buffer A. The ligation reaction was carried out at 37 °C for 1 h in a humid chamber followed by a 10 min and 5 min wash in buffer A. The cells were then incubated with the amplification mix for 2 h at 37 °C in a darkened humidified chamber. After washing with 1 × buffer B (supplied with the kit) for 10 min followed by a 1 min wash with 0.01 × buffer B, the cells were mounted using the mounting media supplied with the kit.

### Statistical analysis

The data are presented as the means ± SEM, and the statistical analyses were performed using SigmaStat software v12 (SPSS, Chicago, IL, USA). The data were analyzed using a one-way analysis of variance and Tukey’s post hoc test; *p* < 0.05 was considered statistically significant.

## Results

### Luteolin elicits cytotoxicity by inducing apoptosis

**Fig. 1:**
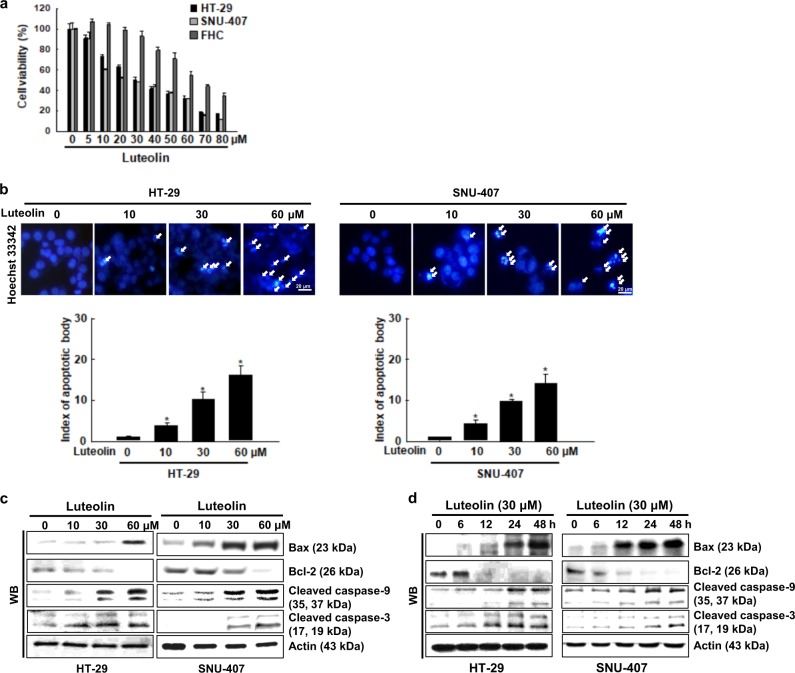
Luteolin elicits cytotoxic effects on human colon cancer cells by inducing apoptosis. **a** HT-29, SNU-407, and FHC cells were treated with various concentrations of luteolin for 48 h, and their viability was assessed using the MTT assay. IC_50_, defined as the concentration that inhibited cell growth by 50%, was determined. **b** Apoptotic body formation was observed using a fluorescence microscope after cells were stained with Hoechst 33342. Arrows indicate apoptotic bodies; *p < 0.05 compared with control cells. Cells were treated with **c** various concentrations of luteolin for 48 h or **d** 30 µM luteolin for various durations. Cell lysates were electrophoresed, and Bax, Bcl-2, active caspase-9, and active caspase-3 proteins were detected using specific antibodies

To investigate the cytotoxicity of luteolin, HT-29, SNU-407 colon cancer cells, and FHC normal colon cells were treated with various concentrations (0–80 μM) of luteolin for 48 h, and their viability was subsequently determined using the MTT assay. Luteolin decreased the viability of all three cell lines in a dose-dependent manner (Fig. 1a). The half-maximal inhibitory concentration (IC_50_) of luteolin was 30 μM in both the HT-29 and SNU-407 cells and 65 μM in the FHC cells, which indicates that the colon cancer cells were more sensitive to luteolin than the normal colon cells. To determine whether luteolin elicits cytotoxicity by inducing apoptosis, the nuclei were stained with Hoechst 33342 and assessed using microscopy. The nuclei were intact in the control cells, whereas luteolin induced nuclear fragmentation, a characteristic of apoptosis, in a dose-dependent manner in the colon cancer cells (Fig. 1b). Moreover, luteolin upregulated the expression of the apoptotic protein Bax, active caspase-9, and active caspase-3, while it downregulated the expression of the anti-apoptotic protein Bcl-2, in a dose- and time-dependent manner (Fig. 1c, d).

### Luteolin promotes ROS scavenging by inducing the expression of antioxidant enzymes

**Fig. 2:**
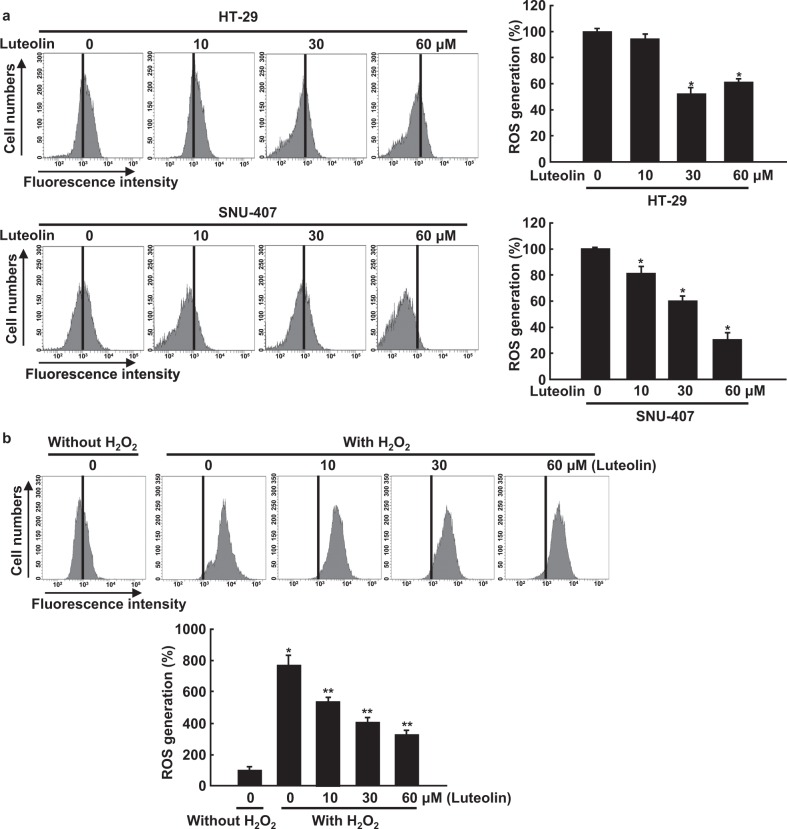

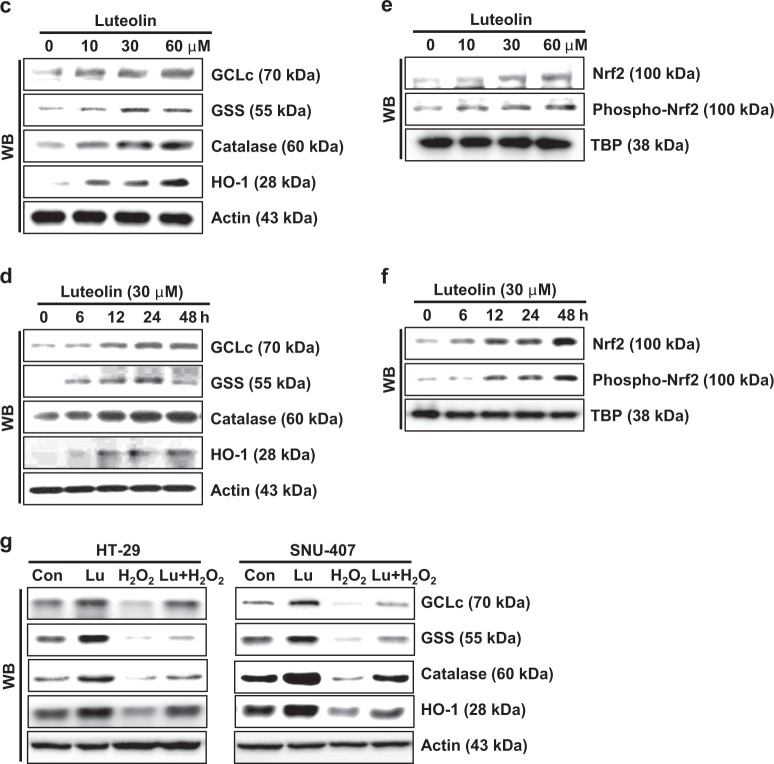
Luteolin promotes ROS scavenging in colon cancer cells by increasing the expression of antioxidant enzymes. **a** Cells were treated with various concentrations (0–60 μM) of luteolin for 24 h, and intracellular ROS were detected using flow cytometry after DCF-DA staining; *p < 0.05 compared with control cells. **b** Cells were pre-treated with various concentrations (0–60 μM) of luteolin for 1 h followed by 80 μM H_2_O_2_ for 24 h. Intracellular ROS were detected using flow cytometry after DCF-DA staining; *p < 0.05 compared with control cells; **p < 0.05 compared with H_2_O_2_-treated cells. Cells were treated with **c**, **e** various concentrations (0–60 μM) of luteolin for 48 h or **d**, **f** 30 μM luteolin for various durations. Western blot analysis was performed using antibodies against GCLc, GSS, catalase, HO-1, Nrf2, and phospho-Nrf2. **g** Cells were treated with 80 μM H_2_O_2_ for 24 h followed by 30 μM luteolin for 48 h, and western blot analysis was performed using antibodies against GCLc, GSS, catalase, and HO-1

We previously reported that luteolin-induced apoptosis is accompanied by scavenging of intracellular and mitochondrial ROS following the activation of antioxidant enzymes^23^. To confirm this, we evaluated intracellular ROS by staining HT-29 and SNU-407 cells with DCF-DA. Flow cytometric analysis demonstrated that the percentage of luteolin-treated cells stained with DCF-DA was significantly lower than the percentage of control cells (Fig. 2a). Luteolin decreased the percentage of cells stained with DCF-DA in a dose-dependent manner up to a concentration of 30 μM. To confirm the ROS-scavenging effect of luteolin, we monitored ROS in HT-29 cells pre-treated with luteolin and then treated with the oxidizing agent H_2_O_2_. Furthermore, pre-treatment with luteolin significantly decreased the percentage of H_2_O_2_-treated cells stained with DCF-DA (Fig. 2b). In addition, luteolin increased the protein expression of the antioxidant enzymes GCLc, GSS, catalase, and HO-1 in a dose- and time-dependent manner (Fig. 2c, d). It also increased the levels of total Nrf2, a critical transcription factor of antioxidant enzymes, and phospho (active)-Nrf2 in a dose- and time-dependent manner (Fig. 2e, f). The assessment of the effect of luteolin on the expression of the antioxidant enzymes indicated that cells treated with H_2_O_2_ alone showed lower expression levels of the antioxidant enzymes, GCLc, GSS, catalase, and HO-1, than the control group; however, the exposure of H_2_O_2_-treated cells to luteolin attenuated this effect (Fig. 2g).

### Luteolin elicits cytotoxicity by inducing DNA demethylation

**Fig. 3:**
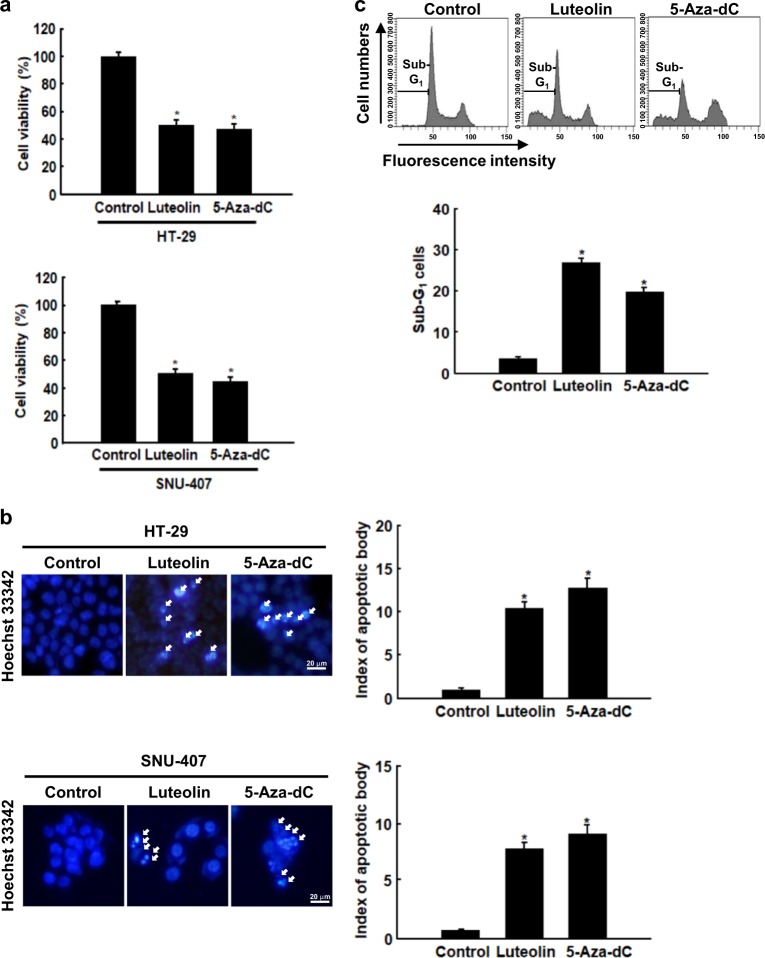
Luteolin exhibits cytotoxicity similar to DNA methylation inhibitor. Cells were treated with 30 μM luteolin or 3.5 μM 5-aza-dC for 48 h. **a** The viability of cells treated with 5-aza-dC and luteolin was determined using the MTT assay; *p < 0.05 compared with control cells. **b** Apoptotic body formation was observed using a fluorescence microscope after cells were stained with Hoechst 33342. Arrows indicate apoptotic bodies; *p < 0.05 compared with control cells. **c** Apoptotic sub-G_1_ DNA content was detected using flow cytometry after the cells were stained with propidium iodide (PI); *p < 0.05 compared with control cells

To determine whether the cytotoxicity of luteolin is related to the regulation of DNA methylation, its effect was compared to that of the DNA methylation inhibitor 5-aza-dC. Similar to luteolin, 5-aza-dC (IC_50_, 3.5 μM) decreased the viability of HT-29 and SNU-407 cells (Fig. 3a). The percentages of cells with fragmented nuclei and in the apoptotic sub-G_1_ phase were significantly higher in the luteolin- and 5-aza-dC-treated cells than in the control cells (Fig. 3b, c). These results suggested that luteolin showed cytotoxicity by inhibiting DNA methylation similar to 5-aza-dC. DNA methylation is catalyzed by three DNMTs, DNMT1, DNMT3A, and DNMT3B, which all add a methyl group to the C5 position of the cytosine ring of the DNA to produce 5-methylcytosine (5-mC). DNMT1 mediates maintenance DNA methylation, whereas DNMT3a and 3b mediate de novo DNA methylation^24^. This methylation process is reversible by DNA demethylases, TET1, TET2, and TET3. These enzymes convert 5-mC to 5-hmC, 5-formylcytosine (5-fC), and 5-carboxylcytosine (5-caC), ultimately generating cytosine^25^.

**Fig. 4:**
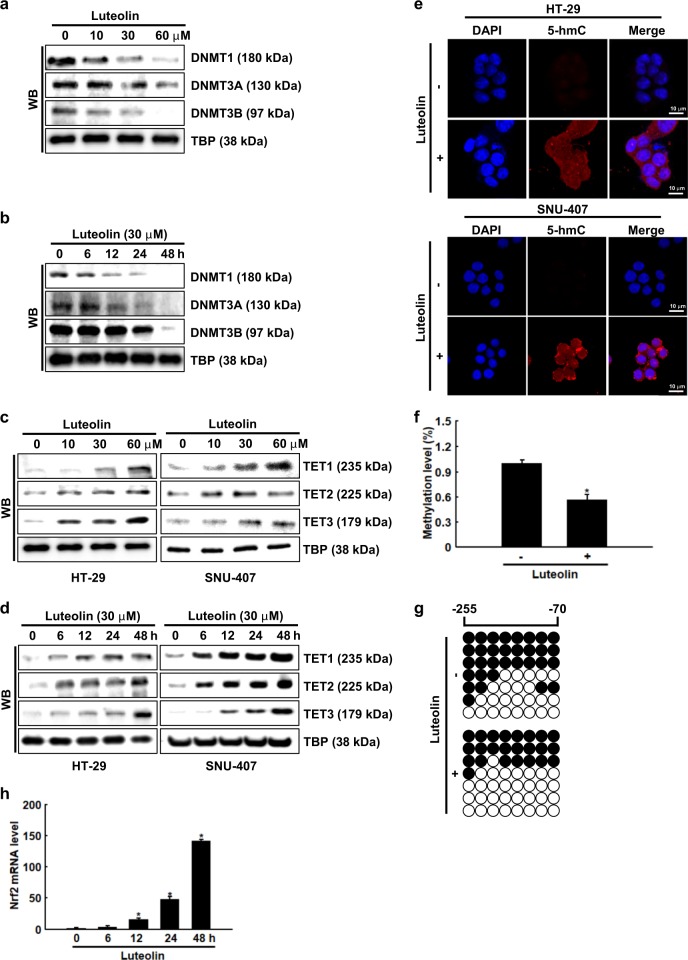
Luteolin exhibits cytotoxicity by inducing epigenetic modifications. Cells were treated with **a**, **c** various concentrations (0–60 μM) of luteolin for 48 h or **b**, **d** 30 μM luteolin for various durations. Western blot analysis was performed using antibodies against DNMT1, DNMT3A, DNMT3B, TET1, TET2, and TET3. **e** Formation of 5-hmC by TETs was assessed using confocal imaging. **f** MS-qPCR analysis of Nrf2 in luteolin-treated cells was performed; *p < 0.05 compared with control cells. **g** Bisulfite sequencing analysis of Nrf2 promoter. Black and white circles represent methylated and unmethylated cytosine residues, respectively. **h** mRNA expression of Nrf2 treated with 30 µM luteolin for 0–48 h was assessed using real-time PCR. *p < 0.05 compared with control cells

Luteolin markedly decreased the protein expression of DNMT1, DNMT3A, and DNMT3B in a dose- and time-dependent manner (Fig. 4a, b). In contrast, it markedly increased the protein expression of TET1, TET2, and TET3 in a dose- and time-dependent manner (Fig. 4c, d). Furthermore, the TET activity was assessed by measuring the 5-hmC levels, which were higher in the luteolin-treated cells than in the control cells (Fig. 4e). Epigenetic modifications of the Nrf2 promoter region contribute to the activation of this gene in cancer^26,27^. The DNA methylation level of the Nrf2 promoter region, as determined using MS-qPCR, was significantly lower in the luteolin-treated cells than in the control cells (Fig. 4f). In addition, the bisulfite sequencing analysis of the Nrf2 promoter region showed that DNA methylation of the Nrf2 promoter region was significantly lower in the luteolin-treated cells (41%) than in the control cells (57%) (Fig. 4g). To investigate the epigenetic mechanism by which luteolin demethylates the Nrf2 promoter and increases Nrf2 transcription, we examined the effect of luteolin on the mRNA expression of Nrf2. Luteolin increased the mRNA expression of Nrf2 colon cancer cells in a time-dependent manner (Fig. 4h).

### TET1 upregulates Nrf2 expression in luteolin-treated cells

**Fig. 5:**
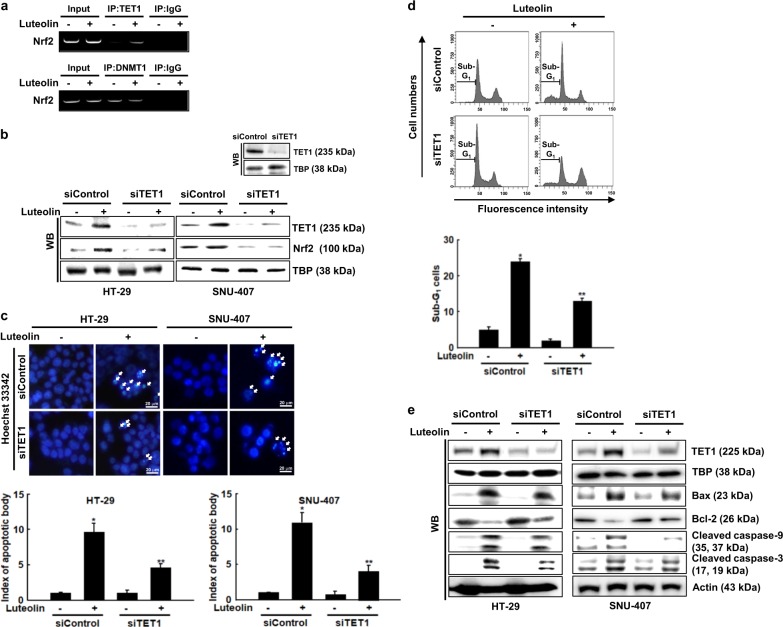
TET1 upregulates Nrf2 in luteolin-treated colon cancer cells. **a** ChIP was performed using anti-TET1 and anti-DNMT1 antibodies and primers designed to amplify the *Nrf2* promoter region. Bands indicate the levels of TET1 and DNMT1 bound to the *Nrf2* promoter region. Inputs represent amplification of total DNA from whole-cell lysates. Cells were transfected with control or TET-targeting siRNA for 24 h. **b** Nrf2 protein expression was determined using western blotting. **c** Apoptotic body formation was observed using a fluorescence microscope after cells were stained with Hoechst 33342. Arrows indicate apoptotic bodies; *p < 0.05 and **p < 0.05 compared with control and luteolin-treated cells, respectively. **d** Apoptotic sub-G_1_ DNA content was detected using flow cytometry after cells were stained with PI; *p < 0.05 and **p < 0.05 compared with control and luteolin-treated cells, respectively. **e** Cell lysates were electrophoresed, and Bax, Bcl-2, active caspase-9, and active caspase-3 proteins were detected using specific antibodies

The results shown in Fig. 4 suggest that the binding ability of DNMTs and TETs to the Nrf2 promoter might be decreased and increased, respectively. To confirm these notions, we performed a ChIP assay. The binding of TET1 and DNMT1 to the Nrf2 promoter markedly increased and decreased, respectively, in the luteolin-treated cells (Fig. 5a). Furthermore, the knockdown of TET1 decreased the protein expression of Nrf2 in the luteolin-treated cells (Fig. 5b). TET1 knockdown decreased the percentage of luteolin-treated cells with fragmented nuclei and cells in the apoptotic sub-G_1_ phase (Fig. 5c, d). In addition, the knockdown of TET1 downregulated the expression of Bax, active (cleaved) caspase-9, and active (cleaved) caspase-3, while it upregulated the expression of Bcl-2 in the luteolin-treated cells (Fig. 5e). These results suggested that TET1 upregulated Nrf2 expression by binding to the Nrf2 promoter and thereby induced apoptosis of the colon cancer cells.

### Nrf2 and p53 interact in luteolin-treated cells

**Fig. 6:**
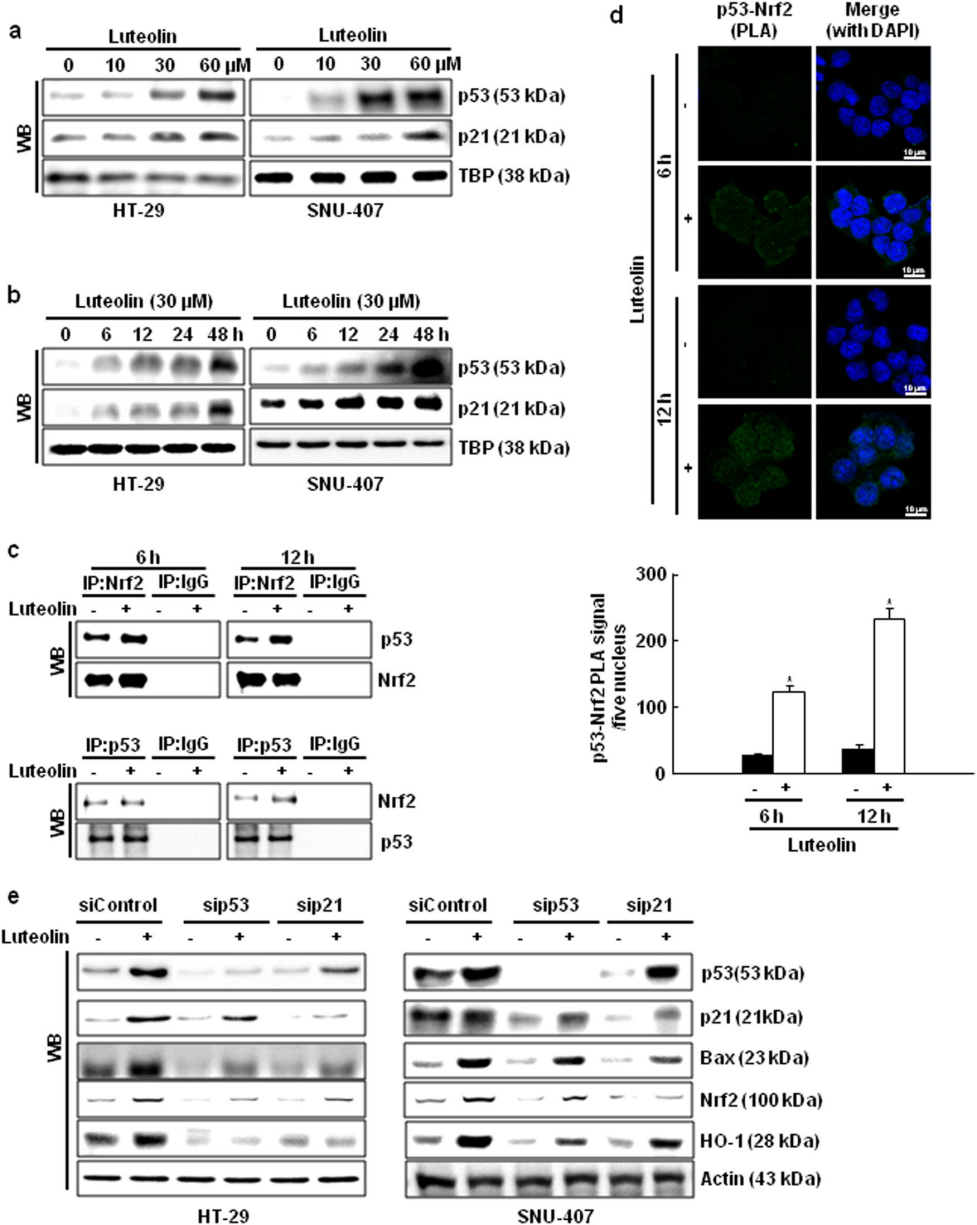
Luteolin increases the interaction between Nrf2 and p53. Cells were incubated with **a** various concentrations of luteolin for 48 h or **b** 30 µM luteolin for various durations. Cell lysates were electrophoresed, and p53 and p21 proteins were detected using specific antibodies. Interaction between p53 and Nrf2 was examined using **c** IP and western blotting with anti-p53 and anti-Nrf2 antibodies and **d** PLA. Each green spot represents a single interaction between p53 and Nrf2. DNA was stained using DAPI; *p < 0.05 compared with control cells. **e** Cells were transfected with p53 or p21-targeting siRNA for 24 h. Bax, Bcl-2, Nrf2, and HO-1 protein expression was determined using western blotting

p53 and Nrf2 both increase the capacity of cells to mitigate oxidative stress and induce apoptosis^28^. Luteolin upregulated the expression of p53 and its target p21 in a dose- and time-dependent manner (Fig. 6a, b). IP showed that complex formation between p53 and Nrf2 was higher in the cells treated with luteolin for 6 or 12 h than in the control cells (Fig. 6c). The analysis of the binding between p53 and Nrf2 using PLA supported this finding (Fig. 6d). Furthermore, the knockdown of p53 and p21 using siRNA transfection decreased the expression levels of Bax, Nrf2, and HO-1; however, the expression level of Bcl-2 increased (Fig. 6e).

## Discussion

The bioflavone luteolin, found in various fruits and vegetables, has been reported to significantly inhibit colon cancer and carcinogenesis by exerting strong antioxidant effects^18,26,29^. In the present study, the IC_50_ of luteolin was lower in the human colon cancer (HT-29 and SNU-407) cells than it was in the human normal colon (FHC) cells, which suggests that colon cancer cells are more sensitive to luteolin than normal colon cells.

A diet rich in fruits and vegetables reduces oxidative stress by increasing antioxidant activity and thereby significantly reduces the risk of colon cancer^30^. ROS accumulate in breast, colon, pancreatic, prostate, and other types of cancer^31,32^. In the present study, luteolin decreased the levels of ROS in colon cancer cells and increased those of antioxidant proteins, including Nrf2.

Various flavonoids exhibit anticancer effects by modulating epigenetic signaling. For example, luteolin suppresses the growth of human prostate cancer cells by inhibiting the activities of DNMTs and EZH2^33^. Moreover, luteolin blocks cell transformation by activating the Nrf2 pathway via downregulation of DNMT and HDAC expression^19^. The anticancer effect of luteolin was similar to that of 5-aza-dC, which inhibits DNA methylation. Furthermore, curcumin, sulforaphane, and 3,3′-diindolylmethane demethylated the Nrf2 promoter and stimulated Nrf2 signaling in the prostate of TRAMP mice and TRAMP C1 cells partly by suppressing the expression of DNMTs and histone deacetylases^34–36^. A curcumin analog restored the expression of Nrf2 and its downstream detoxification enzymes by inducing epigenetic modifications, which led to the suppression of colony formation by prostate cancer cells^37^.

Furthermore, the protein expression of DNMT1, DNMT3A, and DNMT3B decreased and that of the DNA demethylases (TET1, TET2, and TET3) and their activities increased in luteolin-treated cells. In addition, luteolin significantly decreased DNA methylation of the Nrf2 promoter region of the colon cancer cells, which corresponded to an increased mRNA expression of Nrf2 in the luteolin-treated cells. Moreover, TET1 increased Nrf2 expression, whereas knockdown of TET1 decreased Nrf2 expression in luteolin-treated cells. 5-Aza-dC exhibits cytotoxicity on hepatocellular carcinoma cells by activating TETs^38^. The knockdown of TET1 decreased the apoptosis of the luteolin-treated cells.

The transcriptional activation by Nrf2 in response to oxidative stress involves a subset of p53 targets. Recent findings indicate that p21, a p53 target gene, stabilizes Nrf2 by binding to KEAP1 and interfering with its ability to promote the ubiquitylation and proteasomal degradation of Nrf2^39,40^. NQO1, an Nrf2 target, interacts with p53 and blocks its degradation by the 20S proteasome^41,42^. Luteolin upregulated the expression of p53 and its target protein p21 and increased the interaction between Nrf2 and p53, as detected using IP and the PLA. The knockdown of p53 and p21 decreased the expression levels of antioxidant and apoptotic factors, which suggests that p53 and p21 might stabilize Nrf2.

**Fig. 7:**
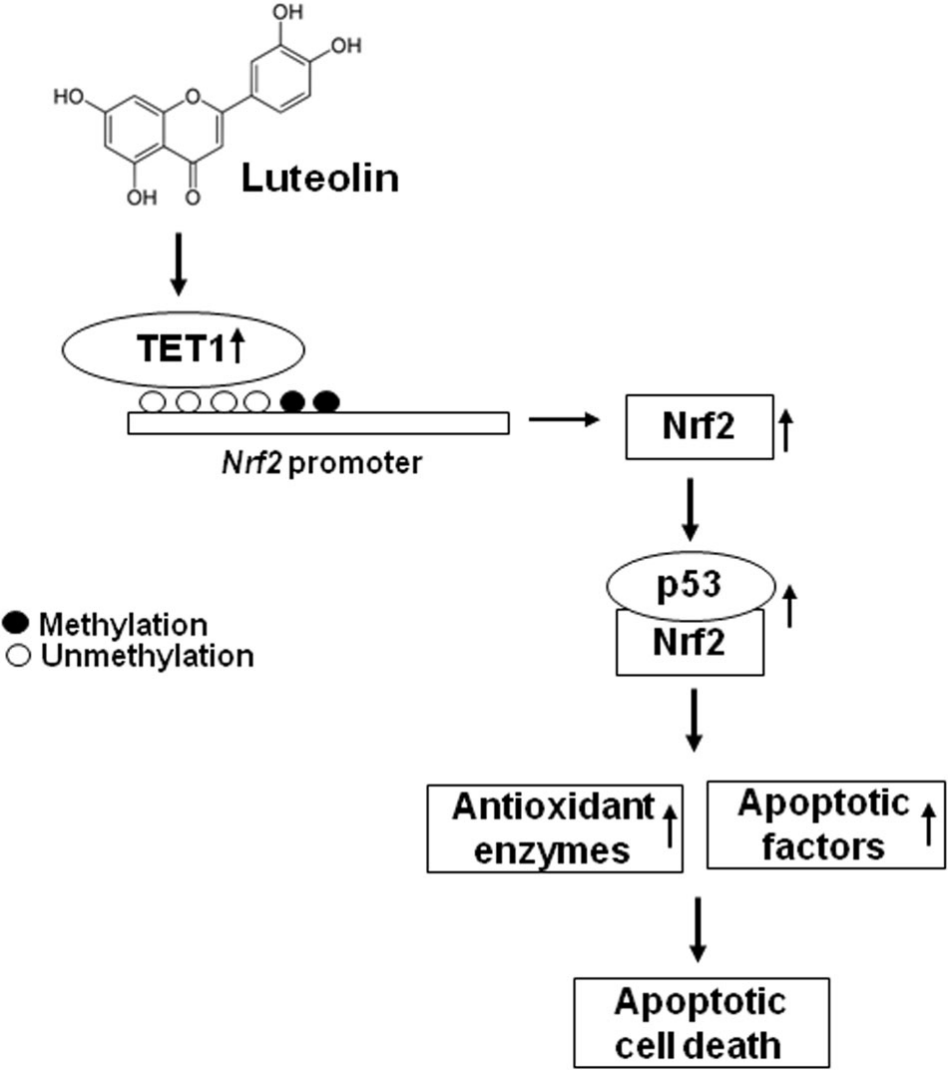
Proposed model of the roles of TET1 and Nrf2 in the antioxidant and apoptotic effects of luteolin. Luteolin increases the expression of the DNA demethylase TET1, which upregulates the expression of Nrf2 by binding to its promoter in colon cancer cells. Upregulation of Nrf2 expression increases the interaction between p53 and Nrf2, which upregulates the expression of antioxidant enzymes and apoptosis-related proteins. Thus, TET1-mediated upregulation of Nrf2 in luteolin-treated colon cancer cells elicits anticancer effects

In conclusion, luteolin exhibited anticancer effects on colon cancer cells by inducing apoptosis (Fig. 7). This effect was dependent on increased transcription of Nrf2 mediated by inducing DNA demethylation of its promoter. In addition, luteolin increased the interaction between Nrf2 and p53, which increased the expression of antioxidant enzymes and apoptosis-related proteins. These findings provide insights into the potential applications of luteolin for the prevention and treatment of cancer.
